# Episodic evolution of a eukaryotic *NADK* repertoire of ancient provenance

**DOI:** 10.1371/journal.pone.0220447

**Published:** 2019-08-01

**Authors:** Oliver Vickman, Albert Erives

**Affiliations:** Department of Biology, University of Iowa, Iowa City, IA, United States of America; Laboratoire Oceanologique de Banyuls sur Mer, FRANCE

## Abstract

NAD kinase (NADK) is the sole enzyme that phosphorylates nicotinamide adenine dinucleotide (NAD+/NADH) into NADP+/NADPH, which provides the chemical reducing power in anabolic (biosynthetic) pathways. While prokaryotes typically encode a single NADK, eukaryotes encode multiple NADKs. How these different *NADK* genes are all related to each other and those of prokaryotes is not known. Here we conduct phylogenetic analysis of *NADK* genes and identify major clade-defining patterns of *NADK* evolution. First, almost all eukaryotic *NADK* genes belong to one of two ancient eukaryotic sister clades corresponding to cytosolic (“cyto”) and mitochondrial (“mito”) clades. Secondly, we find that the cyto-clade *NADK* gene is duplicated in connection with loss of the mito-clade NADK gene in several eukaryotic clades or with acquisition of plastids in Archaeplastida. Thirdly, we find that horizontal gene transfers from proteobacteria have replaced mitochondrial *NADK* genes in only a few rare cases. Last, we find that the eukaryotic cyto and mito paralogs are unrelated to independent duplications that occurred in sporulating bacteria, once in mycelial Actinobacteria and once in aerobic endospore-forming Firmicutes. Altogether these findings show that the eukaryotic *NADK* gene repertoire is ancient and evolves episodically with major evolutionary transitions.

## Introduction

Nicotinamide adenine dinucleotide (NAD) is a dinucleotide cofactor essential to all living cells [1–7]. NAD is composed of one nucleotide featuring the pyrimidine base nicotinamide (N) and another nucleotide featuring the purine base adenine (A). The nicotinamide base of NAD is an electron carrier central to NAD’s function. In the oxidized form, NAD is written as “NAD+” to emphasize it is in the unreduced state. The reduced form occurs after acquisition of two electrons and 1 proton (+ 2e^-^ + H^+^) and is written as “NADH”. NADH is a strong, biologically-relevant, electron donor such that a 1-to-1 mixture of NADH and NAD+ has a redox potential of -320 mV [5]. We can diagram the NAD+/NADH redox pair as follows:

$$\underline{N^{+}}∼1\prime \left(2\prime ∼OH\right)\left(3\prime ∼OH\right)5\prime ∼PO_{3}{}^{‐}–O–PO_{3}{}^{‐}∼5\prime \left(3\prime ∼OH\right)(2\prime ∼OH)1\prime ∼A=NAD+\left[‐1\;net\;charge\right]$$

$$\underline{HN}∼1\prime \left(2\prime ∼OH\right)\left(3\prime ∼OH\right)5\prime ∼PO_{3}{}^{‐}–O–PO_{3}{}^{‐}∼5\prime \left(3\prime ∼OH\right)(2\prime ∼OH)1\prime ∼A=NADH\;\left[‐2\;net\;charge\right]$$

NAD’s adenine nucleotide holds one bit of information read by enzymes that recognize the absence (dot underlined hydroxyl group above) or presence of a phosphate group at the 2' ribose carbon (dot underlined phosphate group below). This phosphorylation site is far removed from the nicotinamide base that serves as the electron-carrying moiety and is thus functionally independent of NAD’s redox state. While NADH is mainly used as a reducing agent in catabolic reactions and in the electron transport chain of oxidative phosphorylation, NADPH is mainly used as a reducing agent in anabolic reactions. We can diagram the oxidized and reduced forms of phosphorylated NAD as follows:

$${\underline{N}}^{+}∼1\prime \left(2\prime ∼OH\right)\left(3\prime ∼OH\right)5\prime ∼PO_{3}{}^{‐}–O–PO_{3}{}^{‐}∼5\prime \left(3\prime ∼OH\right)(2\prime ∼PO_{4}{}^{‐2})1\prime ∼A=NADP+\left[‐3\;net\;charge\right]$$

$$H\underline{N}∼1\prime \left(2\prime ∼OH\right)\left(3\prime ∼OH\right)5\prime ∼PO_{3}{}^{‐}–O–PO_{3}{}^{‐}∼5\prime \left(3\prime ∼OH\right)(2\prime ∼PO_{4}{}^{‐2})1\prime ∼A=NADPH\;\left[‐4\;net\;charge\right]$$

The ability to phosphorylate NAD allows cells to regulate NAD redox balances in two separate pools (+/-PO_3_^-^) for each membrane bound compartment (*e*.*g*., cytosol, mitochondrial matrix, stromal matrix of plastids, and peroxisomes). This enzymatic phosphorylation of NAD+/NADH is carried out exclusively by the NAD kinase (NADK) family. Members of the NADK family can differ by: **(*i*)** substrate specificity for NAD+ and/or NADH; **(*ii*)** donor source of the phosphate group, using either ATP and/or inorganic polyphosphate, poly(P); and **(*iii*)** subcellular localization. NAD(P)H is inferred to have been present in the last universal common ancestor (LUCA) of Bacteria, Archaea, and Eukarya [1–4, 8].

Despite the central role of NADK in the metabolism of cells since LUCA, and despite previous biochemical isolation of this enzymatic activity [9], the genes encoding it in model systems such as *E*. *coli* (*nadK*) and humans (cytosolic *NADK* and mitochondrial *NADK2*) were only identified recently in the post-genomic era [4, 6, 7, 10]. How the human *NADK* and *NADK2* genes are related to other eukaryotic paralogy groups such as the three plant NADK genes [2, 3, 11], and how they are related to the *nadK* genes of various prokaryotic clades has not been determined.

Here, we curate and analyze *NADK* genes from several different eukaryotic and prokaryotic clades to identify the origin of eukaryotic *NADK* genes and their manner of evolution. We find that eukaryotic *NADK* gene evolution is episodic having been sensitive to major evolutionary transitions since the duplication of an *NADK* gene in early eukaryotic evolution. This ancient duplication established two clades of enzymes that ancestrally corresponded to cytosolic (“cyto”-clade) and mitochondrial (“mito”-clade) forms that are maintained in plants, most animals, and some protist groups. Other eukaryotic clades, such as chlorophytes or the clade represented by Holomycota + Amoebozoa have lost their mito-clade gene while duplicating and specializing a second cyto-clade gene for mitochondrial function (“c2m”). In contrast, most other protists have *NADK* genes that cluster in their own lineage-specific cyto-clades. Last, a few protist clades such as Choanoflagellata and Euglenozoa have more recently lost their mito-clade *NADK* gene while gaining an *nadK* gene from α-proteobacteria. Altogether these analyses show that there is a robust phylogenetic signal in the *NADK* sequences and in the particular repertoires of *NADK* genes.

## Results

### An *NADK* gene pair originated early in eukaryotic evolution

**Fig 1:**
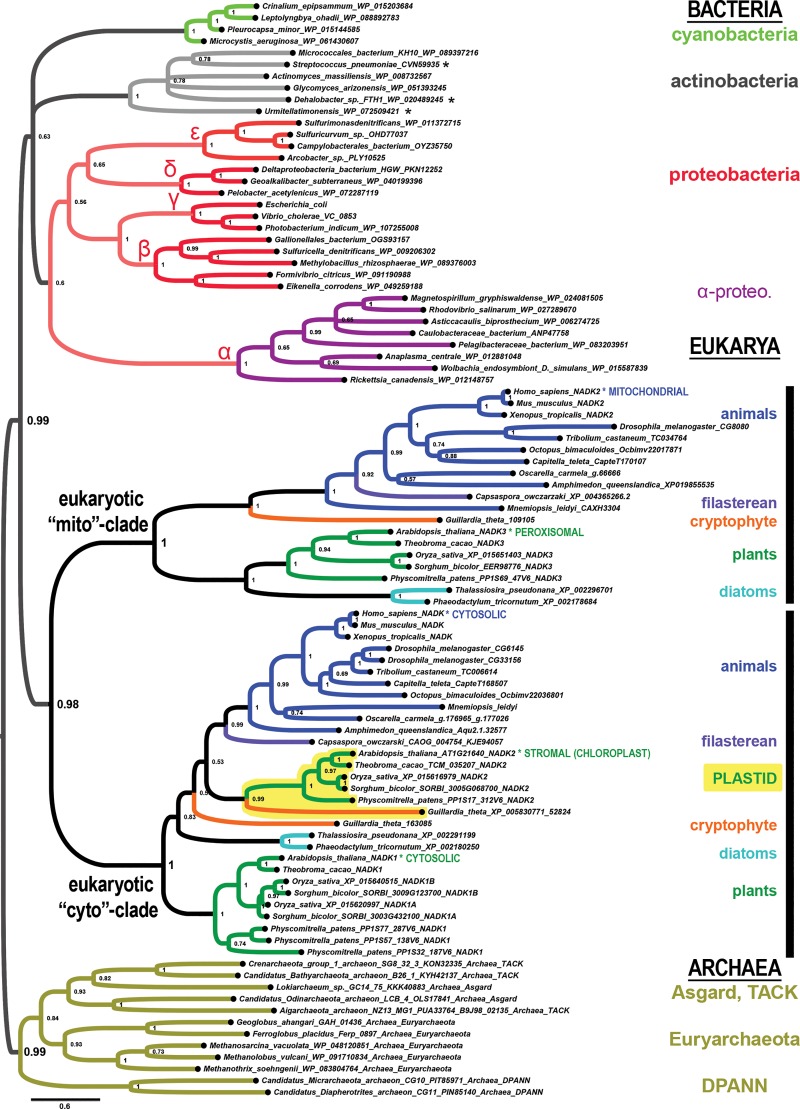
The *NADK* genes of extant eukaryotes originate from two ancient eukaryotic sister clades of uncertain provenance. Shown is a phylogenetic tree computed by Bayesian inference of *NADK* genes from the Bacteria (top clades), Eukarya (middle two clades labeled “mito” and cyto”), and the Archaea (bottom gold clade). Most animals (blue clade), plants (green clade), and a few other protist groups (labeled) have genes from both the mito and cyto subclades. Plants have genes from two cyto-subclades, one of which corresponds to the plant plastid-specific *NADK2* gene. The asterisked labels “MITOCHONDRIAL”, “CYTOSOLIC”, “STROMAL (CHLOROPLAST)” and others indicate lineages with specific experimental data on protein subcellular localization (see text). Node supports in this tree and all other trees represent posterior probabilities. Asterisks indicate sequences from species classified as Firmicutes but which we suspect are lateral gene transfers or misclassifications (also see S1 Fig).

To identify the evolutionary origin of the eukaryotic NADK paralogs, we phylogenetically analyzed diverse eukaryotic groups together with the Archaea, the α-proteobacteria, and the cyanobacteria, which are the proposed origins of the ancestral eukaryotic host cell, its mitochondria, and of plastids, respectively, and additional prokaryotic groups (see Materials & Methods). Our explorations of the NADK tree space identify two ancient eukaryotic *NADK* sister clades of ancient unknown provenance (Fig 1). We refer to these sister clades as the eukaryotic “cyto” and “mito” clades for the inferred ancestral function in an early or stem eukaryote clarity, and for reasons of clarity. This suggested convention bypasses the unrelated NADK numbering systems of vertebrates (*NADK1* and *NADK2*) and plants (*NADK1*, *NADK2*, *NADK3*). For example, human NADK2, which localizes to the mitochondria [10], is not orthologous to *Arabidopsis* NADK2, which localizes to the stromal matrix of chloroplasts [1, 2]; human *NADK2* belongs to the mito-clade while *Arabidopsis NADK2* belongs to one of two plant cyto-subclades. While the main cytosolic enzymes of humans and plants (both named NADK1) belong to the “cyto” clade, the human mitochondrial enzyme NADK2 and the plant peroxisomal enzyme NADK3 belong to the “mito” clade [3, 10] (Fig 1). This can be understood through comparative genomic data that suggests many nuclear-encoded mitochondrial proteins have been secondarily re-targeted to the peroxisome in some eukaryotes [12].

A few major features characterize the ancient eukaryotic configuration of *NADK* genes carried by most plants and animals, and some eukaryotic protists (Fig 1). The first feature is that the ancient eukaryotic nuclear genome encoded two eukaryotic paralogs that are sister clades to each other rather than to any one specific extant prokaryotic clade.

The second major feature is that *NADK* gene duplications predominantly occur in the cyto-clade with only one major exception specific to nematodes, which we describe in a separate section. Some of these duplications are associated with clade-defining evolutionary events (Table 1). For example, land plants and chlorophytes encode a plastid stromal matrix-specific paralog belonging to the cytosolic clade (yellow-highlighted clade in Fig 1). Thus, the chloroplast *NADK2* genes of plants is derived from a duplication of the ancient cyto-clade eukaryotic gene rather than an endosymbiotic transfer from the cyanobacterial progenitor of the plastid.

**Table 1:** Episodic *NADK* duplications associated with clade-defining evolutionary transitions. Rows are ordered from highest to lowest clade levels. Gene duplications identified in a single species are not shown here.

Clade level	Clade	*NADK* gene evolution	Associated event	Proposed driver
Domain	Eukaryota	Duplication and neo-functionalization into cytosolic and mitochondrial *NADK* paralogs (Figs 1 and 2, S1 Fig.)	Endosymbiotic acquisition of mitochondrion	Subcellular compartmentalization
Super kingdom	Archaeplastida	Duplication of cyto-clade *NADK1* and neo-functionalization of plastid *NADK2* (Fig 3)	Endosymbiotic acquisition of plastids	Subcellular compartmentalization
Phylum	Actinobacteria	Duplication and neo-functionalization of *nadK* into *nadK1* and *nadK2* (S1 Fig)	Evolution of mycelial sporulation	Subcellular compartmentalization
Phylum	Nematodes	Unique loss of cytosolic *NADK* and frequent replacement by mitochondrial *NADK2* paralogs (Figs 4 and 5)	Evolution of parasitic micrometazoan	Unknown. Reduced antigenic footprint? Reduced anti-nematodal target?
≥ Class	Kinetoplastida (phylum Euglenozoa)	Acquisition of *nadK* from a stem α-proteobacterial lineage (Fig 6)	Evolution of the kinetoplastid within mitochondrion	Unknown
Class	Choanoflagellates	Horizontal replacement of mito-clade *NADK* with *nadK* from α-proteobacteria (Fig 6)	Unknown	Unknown
Class	Bacilli (phylum Firmicutes)	Duplication and neo-functionalization of *nadK* into *ppnKA* and *ppnkB* (Fig 7, S1 Fig.)	Evolution of aerobic endosporulation	Subcellular compartmentalization
≥ Order	Poales (monocot)	Duplication of *NADK1* into *NADK1A* and *NADK1B* (Figs 1, 2 and 3). Also: [11]	Evolution of triangular protein inclusions in sieve-element plastids	Unknown

The third major feature is that there is no consistent prokaryotic sister clade for the eukaryotic super clade. In our main Fig 1 tree (and other trees), one can see a highly supported Archaea composed of representatives from the Asgard, TACK, Euryarchaeota, and DPANN clades (Fig 1 mustard-highlighted clade). A weakly supported Bacteria features highly-supported clades of representative cyanobacteria (light green), actinobacteria + firmicutes (light gray), and proteobacteria sub-clades (labeled with their Greek letter designations in the red highlighted clades in Fig 1 and remaining figures). Furthermore, the α-proteobacterial *nadK* clade (the so-called purple bacteria), including both free-living (e.g., Rhodospirillales, Caulobacterales, and Pelagibacerales) and endosymbionts (Rickettsiales) is notably long-branched. Their NADK sequences share multiple derived α-proteobacteria-specific changes.

**Fig 2:**
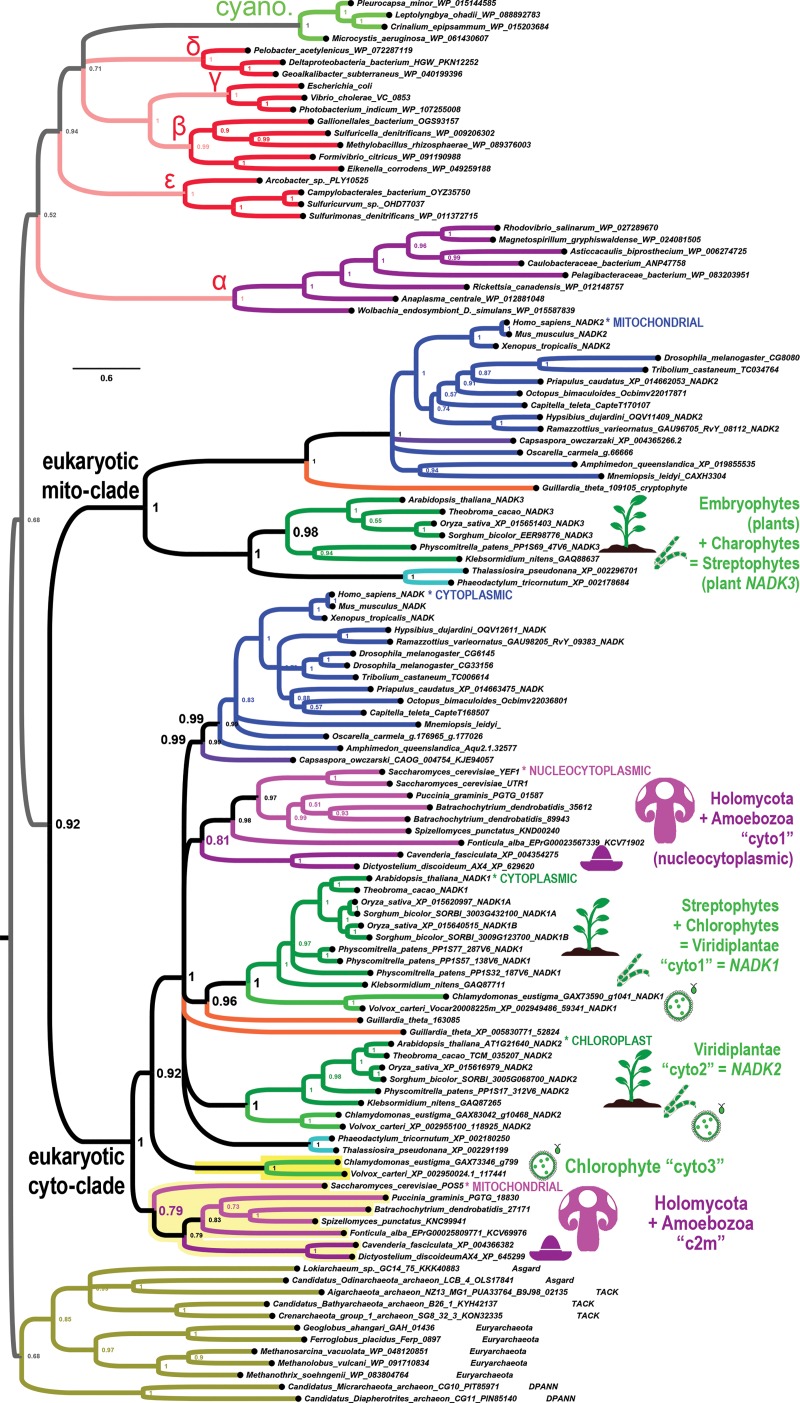
Fungi and Chlorophytes have lost mito-clade NADK genes while independently duplicating a cyto-clade gene. Plants (embryophytes), charophytes, and chlorophytes are each depicted with green icons, while Holomycota (fungi + *Fonticula alba*) and amoebozoans are each depicted with purplish pink icons. A pair each of the fungal groups of ascomycetes, basidiomycetes, and chytridiomycetes were chosen to represent the fungi. The extra cyto-clades for Holomycota + Amoebozoa and for chlorophytes are highlighted in yellow and are found at the bottom of the eukaryotic cyto-subclade.

Some important eukaryotic clades follow a pattern wherein the ancestral mitochondrial NADK paralog is absent and apparently substituted by a neo-functionalized paralogous member of the cyto-clade. The fungal kingdom together with the unranked amoebozoans, and the chlorophyte division of plants (Viridiplantae) are such clades that appear to have lost their mito-clade *NADK* gene in connection with independent duplications of their cyto-clade *NADK* gene (Fig 2, clades highlighted in yellow shading). For example, the chlorophytes *Chlamydomonas* and *Volvox* each have a plant-type *NADK1* gene (cytosolic form) and a plant-like *NADK2* gene (plastid form). However, unlike the embryophytes, including mosses (*Physcomitrella*) and angiosperms (dicots and monocots), and non-embryophyte streptophytes such as the charophyte *Klebsormidium nitens*, chlorophytes lack a detectable mito-clade *NADK* gene (plant *NADK3*). Instead, the chlorophytes have a third “cyto” clade (Fig 2, bright yellow shaded clade).

Similar to the chlorophytes, the fungi and amoebozoans possess a clade of *NADK* genes grouping well within the cyto-clade and a more a basally branching duplicate cyto-clade that is not part of the mito-clade (Fig 2 light yellow shaded clade). In *Saccharomyces cerevisiae*, duplicate genes (*YEF1* and *UTR1*) in the embedded cyto clade correspond to nucleocytoplasmic versions as expected, while the basally branching *POS5* gene corresponds to a known mitochondrial version [13] (“c2m”). Furthermore, the unusual, non-amoebozoan, cellular slime mold, *Fonticula alba*, which together with Fungi represent the Holomycota, appropriately follows the fungal pattern in its *NADK* gene repertoire.

The second basal “c2m” subclade of Holomycota + Amoebozoa is a sister-clade to the entire cyto-clade as opposed to being a sister-clade to the main cyto-clade containing *YEF1* and *UTR1* (Fig 2). An interpretation of the paralogous fungal “c2m” clade requires that Holomycota and Amoebozoa are a monophyletic clade that excludes Holozoa. Or, if instead Holomycota and Holozoa form a monophyletic clade that excludes Amoebozoa, then one has to suppose the more complicated scenario that Holozoa has lost this additional “c2m” gene. Alternatively, these extra cyto-clades might represent divergent clades that have “fallen out” of the eukaryotic mito-clade.

**Fig 3:**
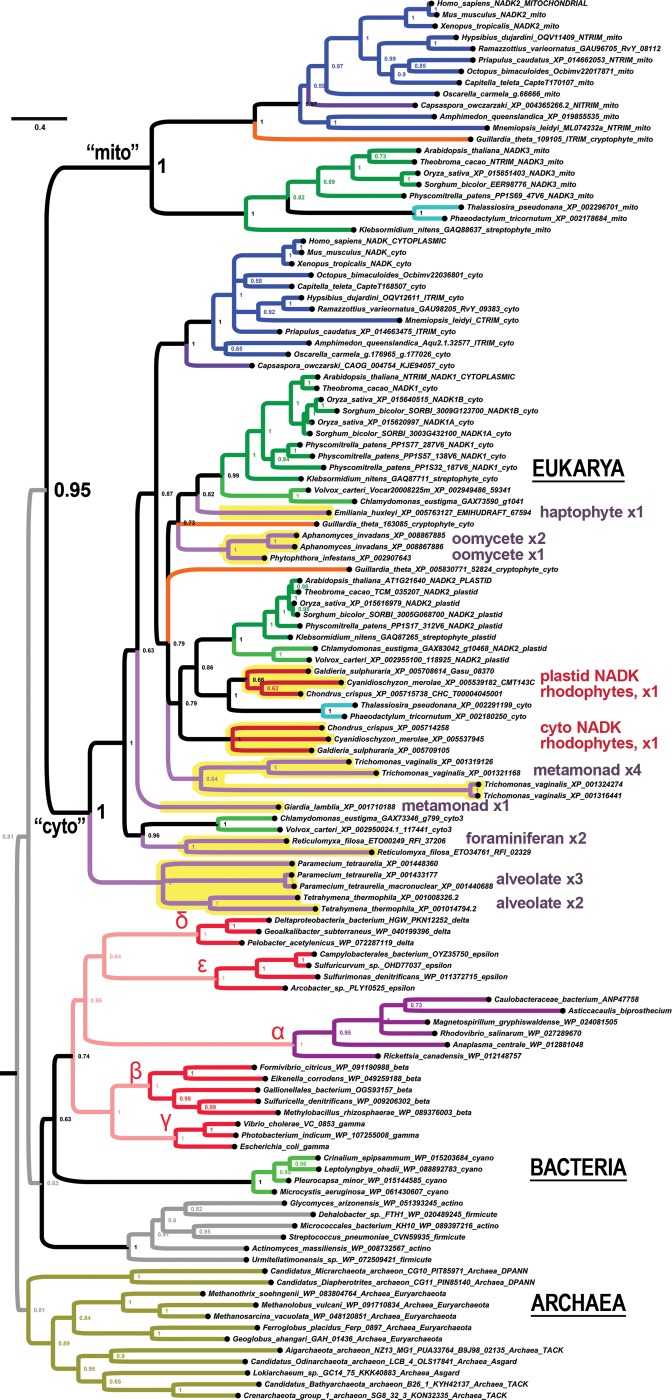
Many eukaryotic protists do not possess the mito-clade *NADK* gene of plants and animals. Unlike fungi, amoebozoans, and chlorophytes, all of which have additional *NADK* cyto-clade genes while missing a mito-clade *NADK* gene, many eukaryotic protist lineages that are also missing mito-clade *NADK* genes possess *NADK* cyto-clade genes that are only recently duplicated, if at all.

We find that other eukaryotic protists harbor only cyto-clade *NADK* genes and frequently these have duplicated in their specific lineages (light purple highlighted clades in Fig 3). Some of these are interesting because of the divergent evolutionary pathways they have taken with regard to their mitochondrion-related organelles [14]. We summarize the results for each separate protist clade.

#### Alveolates

We find lineage-specific duplications for the alveolate *Paramecium tetraurelia*, and these form a sister clade to the lineage-specific duplications of the alveolate *Tetrahymena thermophile* (Fig 3). The alveolate NADK sub-clade is sister to the rest of the eukaryotic cyto clade, all of which is sister to the eukaryotic mito clade.

#### Rhizaria

We find lineage-specific cyto-clade duplications for organisms within two different clades of Rhizaria: the phylum Cercozoa and the subphylum Foraminifera of phylum Retaria. In most trees, the cercozoan *Plasmodiophora brassicae* has a pair of lineage-specific duplications while the foraminiferan *Reticulomyxa filosa* also has a pair of lineage-specific duplications. These two rhizarian typically group together with the third set of cyto-clade genes of chlorophytes, although this is weakly supported (posterior probabilities between 0.51 and 0.83 in different trees).

#### Oomycetes

We find that the oomycete *Aphanomyces invadens* has a lineage-specific pair of duplicated cyto-clade genes that are most closely related to each other. This gene is sister to the single gene found for the oomycete *Phytophthora infestans*.

#### Metamonads

We find that the flagellated trichomonad parasite *Trichomonas vaginalis* has two pairs of duplicated cyto-clade genes for a total of four *NADK* genes. All four group together as three different duplications of different ages (cyto → cyto-A + cyto-B → → cyto-A1 + cyto-A2 and cyto-B1 + cyto-B2; see Fig 3). These gene duplications may be consistent with the finding of one or more large-scale genome duplications in its recent past [15]. In contrast, we find only one *NADK* gene for the flagellated diplomonad parasite *Giardia lamblia* but this is in the same sub-clade (typically) with the *Trichomonas* duplications.

The mitosome of the anaerobic *Giardia* is a derivative mitochondrial relic that no longer has a mitochondrial genome and no longer supports aerobic respiration [14, 16–20]. The only known function of its mitosome is to continue supporting iron-sulfur cluster formation [16, 18, 20]. Thus, the finding of a single cyto-clade *NADK* gene is consistent with the (inferred) loss of its mito-clade *NADK* gene.

*Trichomonas vaginalis* also features a derivative and non-conventional mitochondrial organelle [14]. This double-cell walled organelle has been metabolically reprogrammed as a hydrogenosome that produces molecular hydrogen and ATP from the byproducts of glycolysis in the cytosol. The hydrogenosome continues to support several metabolic synthesis pathways that would require NADPH [21]. Thus, it would make sense that *Trichomonas* continues to harbor multiple genes in contrast to the single gene of *Giardia*.

#### Rhodophytes

Rhodophyta and Viridiplantae together comprise the Archaeplastida, which are supposed to have descended from the same primary endosymbiotic event between a eukaryotic protist and a cyanobacteria. We find that three species of rhodophytes from three genera each have a pair of cyto-clade *NADK* genes grouping into two separate rhodophyte sub-clades (Fig 3). One of these rhodophyte sub-clades is sister to the Stramenopiles cyto-clade and together these are sister to the *NADK2* plastid clade of Viridiplantae. This grouping is thus consistent with Archaeplastida. The second rhodophyte cyto-clade is also sister to the Archaeplastida *NADK2* clade, which would be partially consistent with an ancient cyto duplication into the plant *NADK1* and *NADK2* gene clades.

#### Haptophytes

We find only a single identifiable *NADK* gene for the haptophyte *Emiliania huxleyi* (Fig 3). This gene groups basally with the plant NADK1 clade consistent with acquisition of photosynthesis from a higher-order endosymbiotic event.

### Unique fractal evolution of mito-clade NADK in nematodes

**Fig 4:**
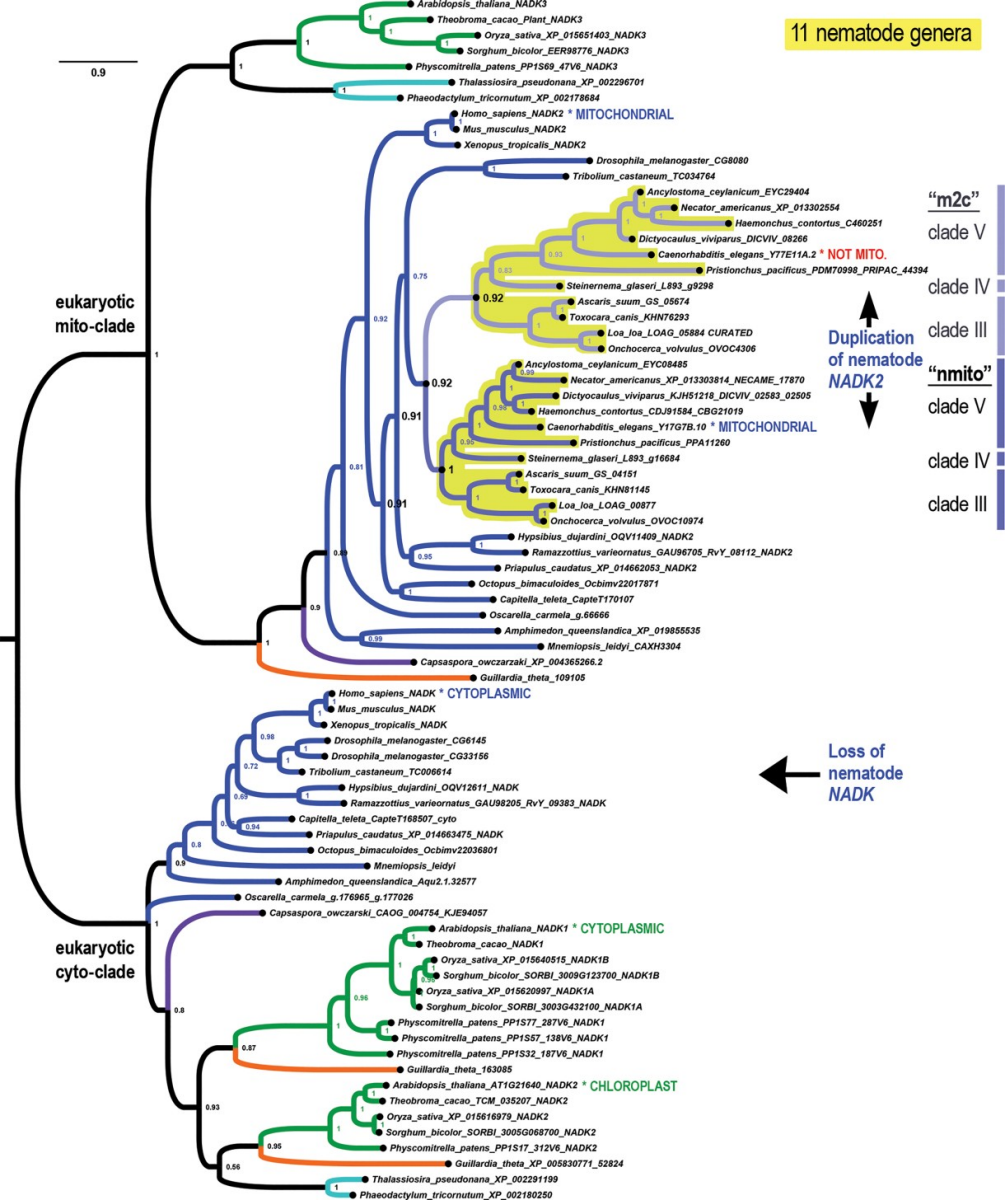
An ancient duplication of *NADK* characterizes Nematoda. All nematodes have missing cyto-clade genes, which was lost in connection with a duplication of the ancestral nematode mito-clade gene. Shown is a phylogenetic tree of *NADK* genes from 11 different nematode genera (highlighted in yellow) that have the ancient nematode duplication of its mito-clade gene into an “nmito” paralog, which encodes an enzyme localized to the mitochondrion in *C*. *elegans*, and an “m2c” paralog, which no longer localizes to the mitochondria in *C*. *elegans*.

**Fig 5:**
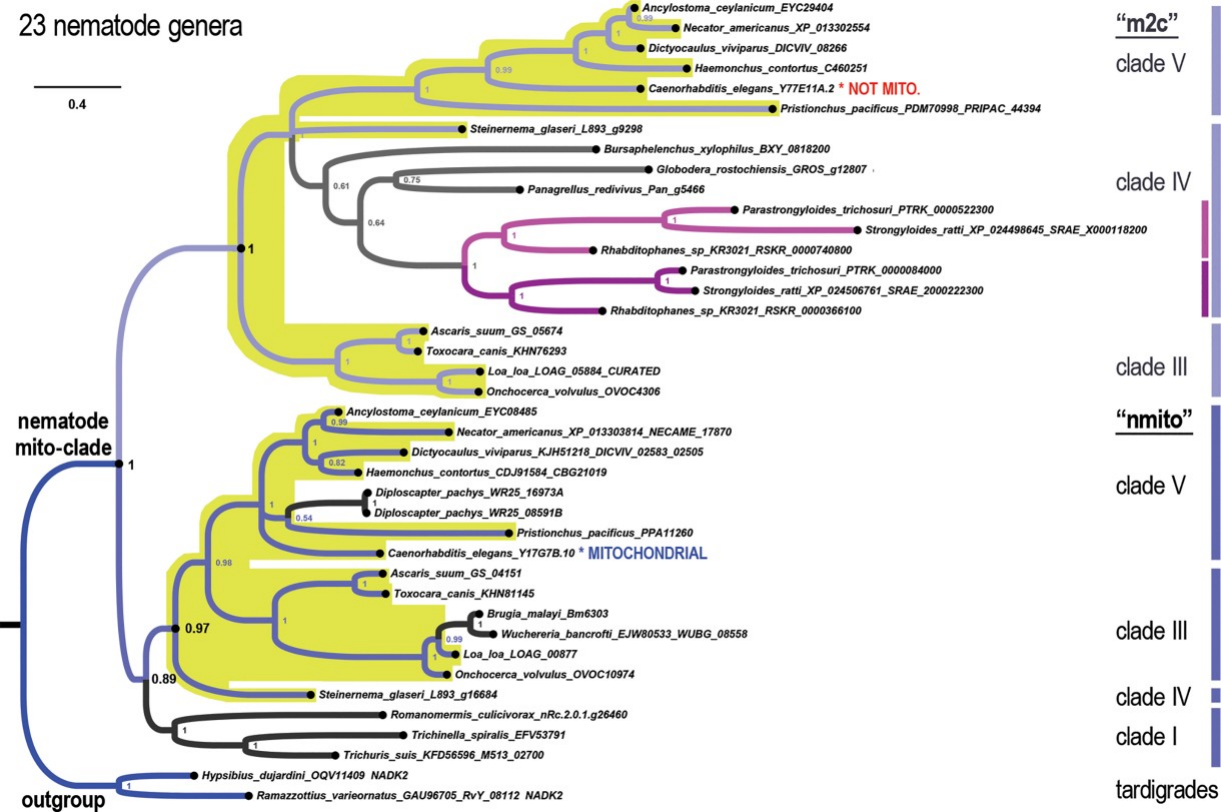
A fractal-like evolutionary pattern characterizes *NADK* evolution in the nematode phylum. Shown is a phylogenetic tree of the nematode mito clade with additional nematode genera that display more recent duplications such as the clade IVb (purplish lineages) in the m2c-subclade and *Diploscapter* genes in the nmito-subclade of clade V nematodes. Other lineages, such as clade I nematodes appear to have only one identifiable gene within one of the two nematode subclades.

We find that the metazoan phylum of nematodes presents a unique pattern *NADK* evolution compared to all other eukaryotes. Whereas we observed all eukaryotes to encode either zero or one mitochondrial *NADK* gene (the “*NADK2*” gene of animals, which is orthologous to the “*NADK3*” gene of plants), the ancestral nematode simultaneously lost its cyto-clade *NADK* gene, while duplicating its mito-clade gene (Fig 4). We can infer that this event occurred specifically within the nematode clade because we are able to find both cyto- and mito- type *NADK* genes in insects (the dipteran *Drosophila* and the coleopteran *Tribolium*) as well as in tardigrade genera (*Hypsibius* and *Ramazzotius*) and in the priapulid worm *Priapulus caudatus* (Fig 4). In contrast, we only find nematode *NADK* genes within the mito-clade (Fig 4 and Fig 5). All of the ecdysozoan mito-clade *NADK* genes, including those of nematodes, form a well-supported sister group to lophotrochozoan genes in a well-supported protostome clade (Fig 4).

Because the slower-evolving *NADK2* paralog of *C*. *elegans* encoded by Y17G7B.10 is known to be mitochondrially-localized while the faster-evolving Y77E11A.2 is not [22], we refer to the genes in the nematode-specific clade containing the former as the “nmito”-subclade (for “nematode mitochondrial”) and the genes in the nematode-specific clade containing the latter as the “m2c”-subclade (for “mito-to-cyto”). The nmito and m2c clades are highly-supported sister-clades in the highly-supported nematode-specific mito-clade (Fig 4 and Fig 5).

Current understanding of nematode phylogeny divides the phylum into a series of numbered clades corresponding to the Dorylaimia (clade I), the Enoplia (clade II), and the Chromadoria (composed of clades III, IV, and V) [23, 24]. Fig 4 depicts *NADK* genes from nematodes in clades III, IV, and V that have retained both the nematode nmito-type and m2c-type genes. In contrast, Fig 5 depicts additional *NADK* genes from nematodes that we observe to have diverged from the ancestral nematode pattern. We now summarize an interesting fractal-like pattern of *NADK* evolution across the various nematode clades for which we had available data (all except clade II/Enoplia). We then speculate on the significance of this evolutionary pattern in the Discussion section.

Clade V (Rhabditina): The overwhelming majority of clade V genera that we analyzed (6/7), and which includes the well-studied *Caenorhabditis elegans* species, have both an nmito- and an m2c- type *NADK* gene (Fig 4). The single exception is for the pair of genes from *Diploscapter*, whose genes represent a lineage-specific duplication of an nmito-type gene along with an inferred loss of its m2c gene (Fig 5).

Clade III (Spirurina): Like clade V, the majority of clade III genera that we analyzed (4/6) have both an nmito- and an m2c- type *NADK* gene (Fig 4). The exception is for the genes from *Brugia malayi* and *Wucheria bancrofti* for which we were only able to find a single nmito-type gene from each. For example, we were not able to find credible matches to m2c-type genes even by using m2c protein sequences to search these genomes using TBLASTN (protein to translated nucleotides) and relaxed parameters (PAM250 and BLOSUM45 substitution matrices with relaxed gap penalties).

Clade IV (Tylenchina): Except for the nmito and m2c genes from *Steinernema glaseri* (Fig 4), the rest of the clade IV sequences, which pertain to 6 additional genera, belong exclusively to the m2c-type clade. Interestingly, three of these genera have a shared ancestral duplication of their m2c gene. Thus, on the basis of this m2c duplication alone, we can phylogenetically place the *Parastrongyloides*, *Strongyloides*, and *Rhabditophanes* genera in a single “IVb” sub-clade. All of the remaining clade IV genera (4/7 genera) branch basally and are not resolved as a single monophyletic clade using just these *NADK* sequences.

Clade I (Dorylaimia): Last, we were only able to find *NADK* genes from the nmito-clade for three genera of clade I nematodes, all of which are highly derived parasitic nematodes of mammals (*Trichinella* and *Trichuris*) or insects (*Romanomermis*) and one of which is an intracellular parasite (*Trichinella*) (Fig 5). These were grouped together with a posterior probability of 1 and as a sister-clade to the remaining nmito genes with a posterior probability of 0.89 (Fig 5). Thus, this topology does not support that these sequences are a sister-clade to a super-clade of nmito and m2c sub-clades, but rather that clade I nematodes lost their m2c genes. Nonetheless, we cannot completely rule out that the ancestral nematode duplication that produced the nmito and m2c paralogs occurred during early nematode divergence.

### Loss of mito-clade *NADK* with horizontally-transferred α-proteobacterial *nadK*

**Fig 6:**
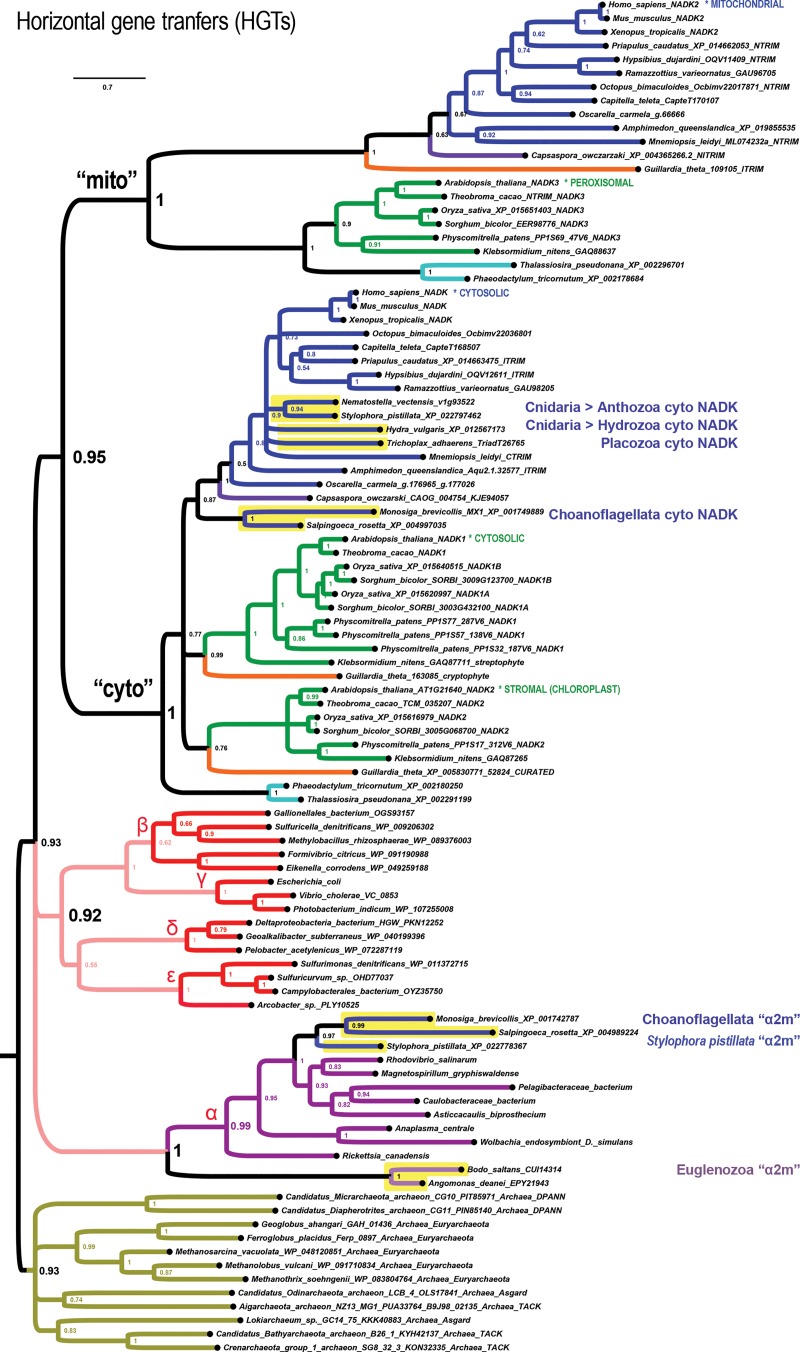
Rarely, eukaryotic lineages replaced mito *NADK* genes with horizontal transfer of α-proteobacterial *nadK*. Choanoflagellates, euglenozoans, and the cnidarian *Stylophora pistillata* eukaryotic mito-clade *NADK* genes while also possessing nadk genes apparently acquired from different lineages of α-proteobacteria. These apparent cases of HGT thus appear to be “α2m” replacements of mito-type NADK.

We find three eukaryotic clades/lineages have lost mito-clade genes while simultaneously gaining genes from different lineages of α-proteobacteria. The first example concerns the choanoflagellates *Monosiga brevicollis* and *Salpingoeca rosetta*, which encode cyto-clade NADK sequences that are most closely related to each other in a holozoan clade that includes metazoans and the filozoan Capsaspora (Fig 6 “choano-cyto”). However, the choanoflagellates lack NADK sequences in the mito subclade (Fig 6 “choano-mito loss”). Instead of having canonical holozoan mito-clade genes, choanoflagellates harbor *nadK* genes horizontally acquired from the α-proteobacteria (Fig 6 “choano-alpha”). That this is a horizontal gene transfer (HGT) is further supported by the presence of amino acid sequences only shared by the α-proteobacteria NadKs. HGT is also implied given the presence and position of the more ancient NADK paralogy groups shown in Fig 1.

We find another apparent case of mito-clade replacement with α-proteobacterial HGT with one of the two anthozoan species of cnidarians, *Stylophora pistillata* (Fig 6). All other cnidarians that we analyzed, including the anthozoan *Nematostella vectensis* and the hydrozoan *Hydra vulgaris* have only a single gene each within the metazoan cyto clade. A similar case of a single cyto *NADK* gene characterizes the placozoan *Trichoplax adhaerens* (Fig 6).

Last, we find that the free-living, euglenozoan excavate *Bodo saltans* and another euglenozoan excavate that is an obligate parasite of some insects, *Angomonas deanei*, have *nadK* genes that form a sister clade with the α-proteobacteria. However, unlike the choanoflagellates and the cnidarian *Stylophora pistillata*, the euglenozoans possess only this single *NADK* gene (Fig 6). Nonetheless, the euglenozoan genes are consistently paired with each other across different trees as are the choanoflagellate choan-alpha genes consistent with these two eukaryotic clades being associated with independent horizontal gene transfers (HGTs) of proteobacterial and α-proteobacterial *nadK*, respectively.

### Independent duplications of *nadK* in sporulating bacteria

We have shown that eukaryotes originated with two ancient *NADK* paralogs of uncertain provenance in relation to prokaryotes. While it has been assumed that prokaryotes typically only have a single *nadK* gene, we desired to test this assumption systematically for comparison to eukaryotes. To investigate whether bacterial and archaeal cells ever have more than one *nadK* gene, we first searched the ComparaEnsembl orthology calls [25]. For example, we identified the number of genes per species on bacteria.ensembl that are orthologous to the single *nadK* gene in *Escherichia coli*. Of the 77 bacterial and 23 archaeal species with *nadK* genes analyzed by EnsemblCompara, only 3 bacterial species have multiple *nadK* genes identified. Two of these species were from the Firmicutes phylum, *Bacillus subtilis* and *Listeria monocytogenes*, while the third was from the actinobacterium *Streptomyces coelicolor*. Each has a pair of *nadK* genes.

**Fig 7:**
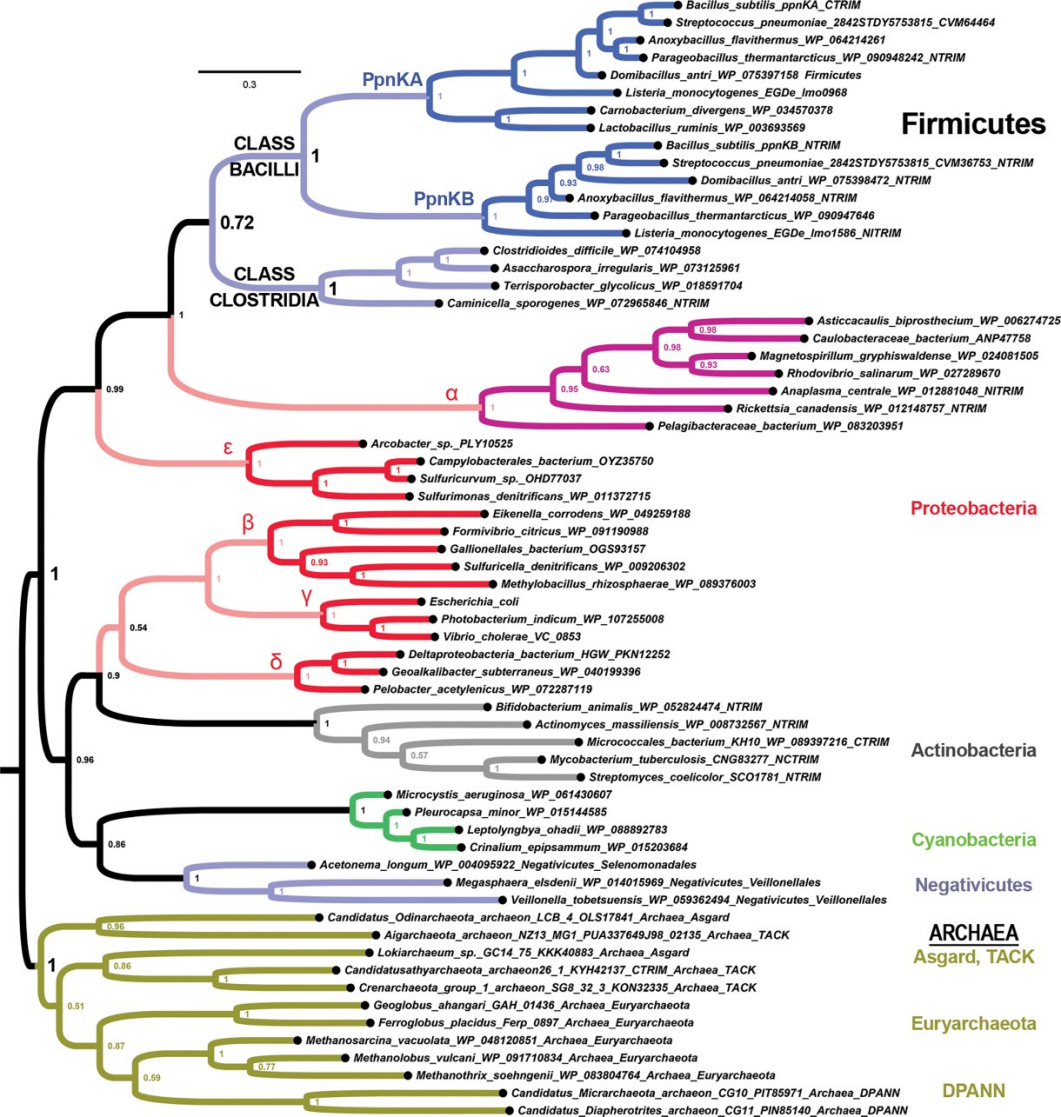
A bacterial duplication of *nadK* in the Firmicutes class of Bacilli. Identification of a duplication of *nadK* into *ppnKA* and *ppnKB* in the occurred after the Bacilli class split with its sister class of Clostridia. The Bacilli class of Firmicutes corresponds to aerobic endospore formers while the Clostridia class corresponds to anaerobic endospore formers. See S1 Fig for a more complete tree that includes the independent duplications in Actinobacteria and Eukaryotes.

To determine the relationships between the gene duplicates of *Bacillus*, *Listeria*, and *Streptomyces*, we computed phylogenetic trees of bacteria using additional species of Firmicutes and Actinobacteria taking care to sample genes coming from the same strains (Fig 7 and S1 Fig). We find that these duplicated *nadK* paralogs stem from two independent duplications in the bacterial phyla of Firmicutes and Actinobacteria (Fig 7 and S1 Fig). For the duplication within Firmicutes, we find that we can place this duplication as occurring within the stem lineage leading to the class Bacilli because the *nadK* genes in the sister class of Clostridia form a sister clade to the Bacilli paralogs (Fig 7). Thus, many species within the class Bacilli have two genes for *polyphosphate/ATP-dependent NAD kinase A* (*ppnKA*) and *ppnKB* as named in *Bacillus subtilis* (Fig 7). The duplication in *Streptomyces* is shared widely within Actinobacteria because the paralogs “*nadK1*” and “*nadK2*” (so named here to distinguish them from the independent duplication in Bacilli) can be found in multiple orders of Actinobacteria: Streptomycetales (*Streptomyces* and *Streptacidiphilus*), Pseudocardiales (*Amycolatopsis*), and Propionibacteriales (*Kribbela*) (S1 Fig).

Current taxonomic classification based on 16S rRNA splits the Firmicutes class Bacilli into the paraphyletic order Bacillales and the monophyletic order Lactobacillales [26]. We find that some Lactobacillales genera (*Lactobacillus* and *Carnobacterium*) appear to have lost *ppnKB* while others (such as *Streptococcus*) have not (Fig 7 and S1 Fig). Thus, this *bona fide* paralogous duplication of *nadK* into *ppnKA* and *ppnKB* occurred in the stem Bacilli lineage after the split from class Clostridia. An alignment of different Bacilli NadKA and NadKB proteins shows that there are several residues throughout the enzyme that are evolutionarily differentiated in the NadKA clade and/or the NadKB clade (S2 Fig). Altogether, our results show that the independent bacterial duplications in the Firmicutes class *Bacilli* (Fig 7 and S1 Fig) and the Actinobacteria phylum (S1 Fig) is unrelated to the ancestral duplication in LECA.

To address whether anything is known about differential regulation of *ppnKA* and *ppnKB* in the well-studied *Bacillus subtilis*, we searched a transcriptomic profile generated over hundreds of conditions [27]. Growth profiles in LB, LB + glucose, and M9 minimal media indicate that *ppnKA* has its highest expression levels during exponential growth when *ppnKB* was having its lowest expression levels, while *ppnkB* was having its highest expression levels during stationary phases when *ppnKA* was having its lowest expression levels [27]. Confluent plate growth, induction of swarming, and sporulation (1 hour post initiation of sporulation) are also separate conditions that caused the highest observed expression levels for *ppnkB* but not *ppnKA* [27]. We discuss the significance of these transcriptomic profiles below.

## Discussion

Our results show that *NADK* genes from all domains of life possess a potent phylogenetic signal in the genes themselves and in the series of duplications associated with major clade-defining events in cellular evolution. We identify at least five major duplications associated with the highest taxonomic levels (Table 1). The first major duplication is one associated with the acquisition of the mitochondrion by the latest eukaryotic common ancestor (LECA). We find that the ancient cytosolic and mitochondrial forms of *NADK* were already present as sister paralogs in LECA. Furthermore, we find that these two ancient eukaryotic paralogs could not be definitively assigned either to proteobacteria or to Archaea and are of unknown provenance. Thus, all we can say is that LECA originated with two ancient *NADK* genes specialized for subcellular compartmentalization. If we assume the α-proteobacterial origin hypothesis for mitochondria [28–30], then we must also conclude that the proto-eukaryotic host *NADK* gene duplicated, and then neo-functionalized and replaced the original mitochondrial *nadK* gene. Alternatively, if the eukaryotic mitochondrion is not descended from proteobacteria, then either the host or endosymbiont *nadK* gene could have duplicated in the stem-eukaryote.

A second major duplication event is associated with the higher order endosymbiotic acquisition of plastids in Archaeplastida (Table 1). This occurred by duplication of the cytosolic *NADK* gene of plants (embryophytes), charophytes, rhodophytes, diatoms, and likely cryptophytes. Thus, despite multiple examples of eukaryotic endosymbioses including the mitochondrial endosymbiosis of LECA, the primary and higher order endosymbioses of plants and cryptophytes, host gene duplication and neofunctionalization has predominated over endosymbiotic gene transfer.

We find other independent duplications at the phylum and sub-phylum (class) level in Bacteria and these are potentially interesting for their relationship to membrane compartmentalization associated with mycelial sporulation (Actinobacteria) or aerobic endosporulation (the Firmicutes class of Bacilli). We discuss these details further below.

Last, a striking duplication pattern is seen in the phylum of nematodes, which are unique among eukaryotes in having lost the main cytosolic NADK gene (Fig 4 and Fig 5). This is the converse situation to many eukaryotic protist clades (Fig 2 and Fig 3) in which the mitochondrial *NADK* gene is lost and apparently replaced with duplications of the cytosolic gene. We discuss this special finding in nematodes and its possible significance in relation to nematode biology next. We also propose gene names for the *Caenorhabditis* and *Drosophila* paralogs, all of which are currently unnamed, based on the paralogy relationships (S1 Table).

We also found three rare groupings of eukaryotic *NADK* genes with α-proteobacteria: (*i*) choanoflagellates (*Monosiga brevicollis* and *Salpingoeca rosetta*), (*ii*) the anthozoan cnidarian *Stylophora pistillata*, and (*iii*) euglenozoans (*Bodo saltans* and *Angomonas deanei*) (Fig 6). One possible explanation for the first two, which represent groupings of genes in addition to standard cyto-clade genes possessed by those species, or for all three, is that they represent the true affinity with the proposed clade of mitochondrial ancestry [28, 29]. However, given that these sequences group within α-proteobacteria uniquely (*i*.*e*., mostly separately) and unlike those of the vast majority of eukaryotic *NADK* genes, we interpret these results as gene replacements by recent horizontal gene transfer from α-proteobacterial endoparasites. Frequent HGT from intracellular endosymbionts from this clade may have masked a more ancient provenance for other genes as well. This latter interpretation would be consistent with evidence discounting the α-proteobacterial hypothesis for mitochondria [31, 32].

### On the unprecedented loss of cytosolic *NADK* in nematodes

Nematode *NADK* evolution is fractal-like in that mito-clade gene duplications accompany cyto clade and mito subclade gene losses at multiple time scales. First, like the inferred ancient duplication of eukaryotes that produced the cyto-clade and mito-clade *NADK* genes, a nematode progenitor underwent an ancestral duplication of its mito-clade gene. This occurred in connection with a loss of its ancestral cyto-clade *NADK*, which is otherwise retained in diverse ecdysozoan phyla that we analyzed. Furthermore, there is evidence that the faster-evolving m2c paralog now serves the cytosolic role, while the slower-evolving nmito paralog retains the mitochondrial role. Of the 23 genera from four nematode sub-clades that we analyzed, 11 genera, or almost half, retain both of the ancestral nematode duplications (nmito and m2c types).

Second, four nematode genera have lost one of the two ancient nematode duplications, while undergoing a duplication of their remaining mito-clade gene. This occurred during clade IV divergence in the sub-clade IVb ancestor and more recently in the *Diploscapter* lineage within clade V. Thus, the majority of nematode genera analyzed (15/23) encode two mito-clade genes related to either an ancestral nematode-, a sub-clade IVb-, or a *Diploscapter*- specific duplication event. No other eukaryotic group besides nematodes has duplicated their mito-clade genes even once.

The nematode turnover pattern of paralogs is unambiguous because the nematode nmito and m2c paralogs are deeply-embedded sister clades that are well-supported within the eukaryotic mito-clade, within Metazoa, and within Ecdysozoa. For this reason it is unlikely to be a long-branch attraction artifact between nematode cyto and mito clades. This interpretation is further supported by lineage-specific nematode duplications continuing to occur within these well-supported sister clades. Thus, the absence of this pattern in other eukaryotic groups suggests perhaps that unknown mechanisms or processes besides compensatory turnover underlie the nematode case.

Nematodes are a micrometazoan phylum specialized for parasitism of multicellular plants, animals, and fungi. Greater than one-half of the named species have such lifestyles and ectoparasitic and endoparasitic forms have repeatedly evolved in different clades and across plant and animal Kingdoms [23]. Endoparasitic forms also include both intra-tissue parasites as well as intracellular parasites (e.g., *Trichinella spiralis* larvae invade mammalian muscle cells). Last, even the non-parasitic forms are frequently found to be commensalists of other animals. Thus, we propose that one hypothesis for this persistent pattern of gene evolution in nematodes is that nematodes evolved stealth antigenic footprints and/or attenuated anti-nematodal targets for host defense pathways [33]. Whatever the case, some specific evolutionary driver likely underlies the unique and recurrent pattern of *NADK* gene loss in nematodes.

### Evolution of membrane compartmentalization in sporulating bacteria

Given the *B*. *subtilis* expression profiles of *ppnkA* (exponential growth) versus *ppnkB* (stationary phase, swarming, confluence, and sporulation), the *nadK* duplication in Firmicutes is highly significant (Fig 7 and S1 Fig). It is thought that the bacterial process of endospore formation arose only once in an ancestor of the Firmicutes, with the class Bacilli representing the aerobic endospore formers and the class Clostridia representing the anaerobic endospore formers [34].

Interestingly, the actinobacteria *Streptomyces* is thought to have evolved a type of sporulation (but not the endospore formation pathways of Bacilli) through independent mechanisms [34], and this also is coincident with a duplicated *nadK* gene in this clade (S1 Fig). In *Streptomyces coelicolor*, *nadK1* appears to be associated with cell growth (anabolic pathways) and sporulating stages in both liquid and (mycelated) solid cultures, while *nadK2* appears to be highest in sporulated spores following the mycelated sporulation in solid cultures [35, 36]. Thus, this duplication in Actinobacteria might also be associated with NADK neofunctionalizations associated with mycelial-type sporulation pathways of Actinobacteria [37].

These results suggest that, like the process of membrane compartmentalization in Eukaryotes, the process of endospore formation or some aspects of sporulation relating to bacterial cell envelopes in general [38], may engender a need for different NadK enzymes. Altogether, these results suggest that eukaryotes may have evolved from a prokaryotic ancestor that evolved duplicate *nadK* genes in connection with membrane-bound compartmentalization associated either with endosymbiont recruitment and/or processes analogous to bacterial sporulation.

Last, one outstanding aspect of our results is the persistent affinity between the NadK sequences of Firmicutes and α-proteobacteria, which frequently are sister-clades (*e*.*g*., Fig 7 and S1 Fig). One likely possibility is that this is the result of long-branch attraction. However, an alternative explanation not yet ruled out is that an α-proteobacterial ancestor acquired an NadK-encoding gene from the stem-Firmicutes lineage. In any case, our results indicate that the origin of the paralogous eukaryotic NADK cyto and mito clades are nonetheless of ancient and unknown provenance. Further insights may be possible by integrating our results on the deep phylogeny of the NAD kinase family with both previous and ongoing studies on the evolution of NAD biosynthetic pathways [39, 40], the evolution of NAD-utilizing enzymes [41], and the evolution of life itself [42].

## Materials and methods

### Protein alignment

We constructed amino acid alignments using the MUSCLE algorithm option the MEGA7 program [43, 44]. Unique extensions in the N- and C- termini or unique internal loop insertions were trimmed. In the FASTA headers such sequences are labeled “NTRIM”, “CTRIM”, and “ITRIM” or combinations thereof (*e*.*g*., “NICTRIM”). We also extended partial automatic (hypothetical) gene predictions by consulting the genomic sequence. These sequences are typically labeled “CURATED” after the gene prediction ID in the FASTA header. For alignment, we mostly used the MEGA7 MUSCLE default parameters with adjustments in the gap existence and gap extension parameters. In preliminary explorations of tree space, while the NADK protein data set was being built up, the gap existence and gap extension parameters were left at default (-2.8 and 0.0, respectively). In later refinement of tree space, these were increasingly adjusted as follows. To adjust the ragged N-terminal and C-terminal prokaryotic subset of the data, the gap existence was adjusted down to -1.0. After aligning and trimming these sequences, these were then realigned with the full data set (*i*.*e*., with the eukaryotic clades included) using a gap existence of -2.0 down to -1.8 or -1.5 (depending on the taxa) and a gap extension of -0.02. The specific values used will be listed for each representative tree depicted in the study.

### Phylogenetic analysis

All phylogenetic analyses were conducted using the 64 bit serial version of MrBayes on different Windows machines [45–48]. Metropolis coupling (MC-MCMC) with two “nchains”, a temperature of 0.08, and double precision operations (BEAGLE library) were all enabled. In early analyses, we tested mixed protein substitution models with 25% burn-in generations in prokaryotic + eukaryotic trees, and in prokaryotic-only and eukaryotic-only trees, but as we kept getting the WAG substitution model in later analyses, we switched to using a fixed (WAG) substitution model and gamma-shaped frequency model with some invariant residues (“invgamma”)[49]. Convergence of runs was evaluated by the average standard deviation of split frequencies, and the trees shown had values less than 0.01 (unless otherwise stated) after 300k to 2 M generations (typical run was 1.2 M generations long). Trees were rendered and decorated using the FigTree 1.4.3 tree figure drawing tool.

## Supporting information

S1 Fig The eukaryotic cyto and mito paralogs are independent duplications from those in the phylum Actinobacteria (orange) and the class Bacilli (phylum Firmicutes, mauve). (TIF)

S2 Fig *nadK* paralogs in class Bacilli. The amino acid residues that are differentiated in PpnKA or PpnKB are indicated in blue and red, respectively, while residues that are widely conserved are indicated in yellow.
(TIF)

S1 File NADK data set (sequence files). See S2 Table for a list of files.
(ZIP)

S1 Table Proposed names for *NADK* genes in *Drosophila* and *Caenorhabditis*. (DOCX)

S2 Table List of supplementary files associated with phylogenetic analyses. For each analysis shown, we include the FASTA file (“.fas”), the sequence alignment file (“.masx”), the nexus file (“.nexus”), and associated run files. Each set of files shares the same base file name indicated in the table. Line breaks in FASTA sequences typically indicate places where sequences were trimmed. Some hand curated sequences are documented in WORD files with the base name “CURATION”. All file are included as a compressed archived (“S1 File.tar”).
(DOCX)

## References

[1] Chai MF, Chen QJ, An R, Chen YM, Chen J, Wang XC. NADK2, an *Arabidopsis* chloroplastic NAD kinase, plays a vital role in both chlorophyll synthesis and chloroplast protection. Plant Mol Biol. 2005;59(4):553–64. Epub 2005/10/26. 10.1007/s11103-005-6802-y . 16244906

[2] Hashida SN, Takahashi H, Uchimiya H. The role of NAD biosynthesis in plant development and stress responses. Ann Bot. 2009;103(6):819–24. Epub 2009/02/10. 10.1093/aob/mcp019 19201765 PMC2707885

[3] Waller JC, Dhanoa PK, Schumann U, Mullen RT, Snedden WA. Subcellular and tissue localization of NAD kinases from *Arabidopsis*: compartmentalization of de novo NADP biosynthesis. Planta. 2010;231(2):305–17. Epub 2009/11/19. 10.1007/s00425-009-1047-7 . 19921251

[4] Zhang R. MNADK, a Long-Awaited Human Mitochondrion-Localized NAD Kinase. J Cell Physiol. 2015;230(8):1697–701. Epub 2015/02/03. 10.1002/jcp.24926 . 25641397

[5] Alberts B. Molecular biology of the cell Sixth edition ed. New York, NY: Garland Science, Taylor and Francis Group; 2015 1 volume (various pagings) p.

[6] Kawai S, Mori S, Mukai T, Hashimoto W, Murata K. Molecular characterization of Escherichia coli NAD kinase. Eur J Biochem. 2001;268(15):4359–65. Epub 2001/08/08. 10.1046/j.1432-1327.2001.02358.x . 11488932

[7] Lerner F, Niere M, Ludwig A, Ziegler M. Structural and functional characterization of human NAD kinase. Biochem Biophys Res Commun. 2001;288(1):69–74. Epub 2001/10/12. 10.1006/bbrc.2001.5735 . 11594753

[8] Weiss MC, Sousa FL, Mrnjavac N, Neukirchen S, Roettger M, Nelson-Sathi S, et al The physiology and habitat of the last universal common ancestor. Nat Microbiol. 2016;1(9):16116 Epub 2016/08/27. 10.1038/nmicrobiol.2016.116 . 27562259

[9] McGuinness ET, Butler JR. NAD+ kinase—a review. Int J Biochem. 1985;17(1):1–11. Epub 1985/01/01. . 2987053 10.1016/0020-711x(85)90079-5

[10] Ohashi K, Kawai S, Murata K. Identification and characterization of a human mitochondrial NAD kinase. Nat Commun. 2012;3:1248 Epub 2012/12/06. 10.1038/ncomms2262 23212377 PMC3535332

[11] Li BB, Wang X, Tai L, Ma TT, Shalmani A, Liu WT, et al NAD Kinases: Metabolic Targets Controlling Redox Co-enzymes and Reducing Power Partitioning in Plant Stress and Development. Front Plant Sci. 2018;9:379 Epub 2018/04/18. 10.3389/fpls.2018.00379 29662499 PMC5890153

[12] Gabaldon T, Snel B, van Zimmeren F, Hemrika W, Tabak H, Huynen MA. Origin and evolution of the peroxisomal proteome. Biol Direct. 2006;1:8 Epub 2006/03/25. 10.1186/1745-6150-1-8 16556314 PMC1472686

[13] Ando T, Ohashi K, Ochiai A, Mikami B, Kawai S, Murata K. Structural determinants of discrimination of NAD+ from NADH in yeast mitochondrial NADH kinase Pos5. J Biol Chem. 2011;286(34):29984–92. Epub 2011/07/07. 10.1074/jbc.M111.249011 21730068 PMC3191039

[14] Makiuchi T, Nozaki T. Highly divergent mitochondrion-related organelles in anaerobic parasitic protozoa. Biochimie. 2014;100:3–17. Epub 2013/12/10. 10.1016/j.biochi.2013.11.018 . 24316280

[15] Carlton JM, Hirt RP, Silva JC, Delcher AL, Schatz M, Zhao Q, et al Draft genome sequence of the sexually transmitted pathogen Trichomonas vaginalis. Science. 2007;315(5809):207–12. Epub 2007/01/16. 10.1126/science.1132894 17218520 PMC2080659

[16] Tovar J, Leon-Avila G, Sanchez LB, Sutak R, Tachezy J, van der Giezen M, et al Mitochondrial remnant organelles of Giardia function in iron-sulphur protein maturation. Nature. 2003;426(6963):172–6. Epub 2003/11/14. 10.1038/nature01945 . 14614504

[17] Dolezal P, Smid O, Rada P, Zubacova Z, Bursac D, Sutak R, et al *Giardia* mitosomes and trichomonad hydrogenosomes share a common mode of protein targeting. Proc Natl Acad Sci U S A. 2005;102(31):10924–9. Epub 2005/07/26. 10.1073/pnas.0500349102 16040811 PMC1182405

[18] Jedelsky PL, Dolezal P, Rada P, Pyrih J, Smid O, Hrdy I, et al The minimal proteome in the reduced mitochondrion of the parasitic protist *Giardia intestinalis*. PLoS One. 2011;6(2):e17285 Epub 2011/03/11. 10.1371/journal.pone.0017285 21390322 PMC3044749

[19] Williams CF, Millet CO, Hayes AJ, Cable J, Lloyd D. Diversity in mitochondrion-derived organelles of the parasitic diplomonads *Spironucleus* and *Giardia*. Trends Parasitol. 2013;29(7):311–2. Epub 2013/05/28. 10.1016/j.pt.2013.04.004 . 23706269

[20] Martincova E, Voleman L, Pyrih J, Zarsky V, Vondrackova P, Kolisko M, et al Probing the Biology of *Giardia intestinalis* Mitosomes Using In Vivo Enzymatic Tagging. Mol Cell Biol. 2015;35(16):2864–74. Epub 2015/06/10. 10.1128/MCB.00448-15 26055323 PMC4508323

[21] Huang KY, Ong SC, Wu CC, Hsu CW, Lin HC, Fang YK, et al Metabolic reprogramming of hydrogenosomal amino acids in Trichomonas vaginalis under glucose restriction. J Microbiol Immunol Infect. 2017 Epub 2017/12/05. 10.1016/j.jmii.2017.10.005 . 29198954

[22] Li J, Cai T, Wu P, Cui Z, Chen X, Hou J, et al Proteomic analysis of mitochondria from *Caenorhabditis elegans*. Proteomics. 2009;9(19):4539–53. Epub 2009/08/12. 10.1002/pmic.200900101 . 19670372

[23] Blaxter M, Koutsovoulos G. The evolution of parasitism in Nematoda. Parasitology. 2015;142 Suppl 1:S26–39. Epub 2014/06/26. 10.1017/S0031182014000791 24963797 PMC4413787

[24] Meldal BH, Debenham NJ, De Ley P, De Ley IT, Vanfleteren JR, Vierstraete AR, et al An improved molecular phylogeny of the Nematoda with special emphasis on marine taxa. Mol Phylogenet Evol. 2007;42(3):622–36. Epub 2006/11/07. 10.1016/j.ympev.2006.08.025 . 17084644

[25] Vilella AJ, Severin J, Ureta-Vidal A, Heng L, Durbin R, Birney E. EnsemblCompara GeneTrees: Complete, duplication-aware phylogenetic trees in vertebrates. Genome Res. 2009;19(2):327–35. Epub 2008/11/26. 10.1101/gr.073585.107 19029536 PMC2652215

[26] Boone DR, Castenholz RW, Garrity GM. Bergey's manual of systematic bacteriology. 2nd ed New York: Springer; 2001.

[27] Nicolas P, Mader U, Dervyn E, Rochat T, Leduc A, Pigeonneau N, et al Condition-dependent transcriptome reveals high-level regulatory architecture in *Bacillus subtilis*. Science. 2012;335(6072):1103–6. Epub 2012/03/03. 10.1126/science.1206848 . 22383849

[28] Wang Z, Wu M. An integrated phylogenomic approach toward pinpointing the origin of mitochondria. Sci Rep. 2015;5:7949 Epub 2015/01/23. 10.1038/srep07949 25609566 PMC4302308

[29] Roger AJ, Munoz-Gomez SA, Kamikawa R. The Origin and Diversification of Mitochondria. Curr Biol. 2017;27(21):R1177–R92. Epub 2017/11/08. 10.1016/j.cub.2017.09.015 . 29112874

[30] Yang D, Oyaizu Y, Oyaizu H, Olsen GJ, Woese CR. Mitochondrial origins. Proc Natl Acad Sci U S A. 1985;82(13):4443–7. Epub 1985/07/01. 10.1073/pnas.82.13.4443 3892535 PMC391117

[31] Martijn J, Vosseberg J, Guy L, Offre P, Ettema TJG. Deep mitochondrial origin outside the sampled alphaproteobacteria. Nature. 2018;557(7703):101–5. Epub 2018/04/27. 10.1038/s41586-018-0059-5 . 29695865

[32] Gray MW. Mitochondrial evolution. Cold Spring Harb Perspect Biol. 2012;4(9):a011403 Epub 2012/09/07. 10.1101/cshperspect.a011403 22952398 PMC3428767

[33] Erives AJ. Genes conserved in bilaterians but jointly lost with Myc during nematode evolution are enriched in cell proliferation and cell migration functions. Dev Genes Evol. 2015;225(5):259–73. Epub 2015/07/16. 10.1007/s00427-015-0508-1 26173873 PMC4568025

[34] de Hoon MJ, Eichenberger P, Vitkup D. Hierarchical evolution of the bacterial sporulation network. Curr Biol. 2010;20(17):R735–45. Epub 2010/09/14. 10.1016/j.cub.2010.06.031 20833318 PMC2944226

[35] Yague P, Rodriguez-Garcia A, Lopez-Garcia MT, Rioseras B, Martin JF, Sanchez J, et al Transcriptomic analysis of liquid non-sporulating *Streptomyces coelicolor* cultures demonstrates the existence of a complex differentiation comparable to that occurring in solid sporulating cultures. PLoS One. 2014;9(1):e86296 Epub 2014/01/28. 10.1371/journal.pone.0086296 24466012 PMC3897704

[36] Yague P, Rodriguez-Garcia A, Lopez-Garcia MT, Martin JF, Rioseras B, Sanchez J, et al Transcriptomic analysis of *Streptomyces coelicolor* differentiation in solid sporulating cultures: first compartmentalized and second multinucleated mycelia have different and distinctive transcriptomes. PLoS One. 2013;8(3):e60665 Epub 2013/04/05. 10.1371/journal.pone.0060665 23555999 PMC3610822

[37] Chandra G, Chater KF. Developmental biology of *Streptomyces* from the perspective of 100 actinobacterial genome sequences. FEMS Microbiol Rev. 2014;38(3):345–79. Epub 2013/10/30. 10.1111/1574-6976.12047 24164321 PMC4255298

[38] Tocheva EI, Ortega DR, Jensen GJ. Sporulation, bacterial cell envelopes and the origin of life. Nat Rev Microbiol. 2016;14(8):535–42. Epub 2017/02/25. 10.1038/nrmicro.2016.85 28232669 PMC5327842

[39] Rongvaux A, Andris F, Van Gool F, Leo O. Reconstructing eukaryotic NAD metabolism. Bioessays. 2003;25(7):683–90. Epub 2003/06/20. 10.1002/bies.10297 . 12815723

[40] Gazzaniga F, Stebbins R, Chang SZ, McPeek MA, Brenner C. Microbial NAD metabolism: lessons from comparative genomics. Microbiol Mol Biol Rev. 2009;73(3):529–41, Table of Contents. Epub 2009/09/02. 10.1128/MMBR.00042-08 19721089 PMC2738131

[41] Lesk AM. NAD-binding domains of dehydrogenases. Curr Opin Struct Biol. 1995;5(6):775–83. Epub 1995/12/01. . 8749365 10.1016/0959-440x(95)80010-7

[42] Fontecilla-Camps JC. Geochemical Continuity and Catalyst/Cofactor Replacement in the Emergence and Evolution of Life. Angew Chem Int Ed Engl. 2019;58(1):42–8. Epub 2018/09/01. 10.1002/anie.201808438 . 30168638

[43] Edgar RC. MUSCLE: a multiple sequence alignment method with reduced time and space complexity. BMC Bioinformatics. 2004;5:113 Epub 2004/08/21. 10.1186/1471-2105-5-113 15318951 PMC517706

[44] Kumar S, Stecher G, Tamura K. MEGA7: Molecular Evolutionary Genetics Analysis Version 7.0 for Bigger Datasets. Mol Biol Evol. 2016;33(7):1870–4. Epub 2016/03/24. 10.1093/molbev/msw054 . 27004904 PMC8210823

[45] Huelsenbeck JP, Ronquist F. MRBAYES: Bayesian inference of phylogenetic trees. Bioinformatics. 2001;17(8):754–5. Epub 2001/08/29. 10.1093/bioinformatics/17.8.754 . 11524383

[46] Ronquist F, Huelsenbeck JP. MrBayes 3: Bayesian phylogenetic inference under mixed models. Bioinformatics. 2003;19(12):1572–4. Epub 2003/08/13. 10.1093/bioinformatics/btg180 . 12912839

[47] Ronquist F, Teslenko M, van der Mark P, Ayres DL, Darling A, Hohna S, et al MrBayes 3.2: efficient Bayesian phylogenetic inference and model choice across a large model space. Syst Biol. 2012;61(3):539–42. Epub 2012/02/24. 10.1093/sysbio/sys029 22357727 PMC3329765

[48] Ling C, Hamada T, Bai J, Li X, Chesters D, Zheng W, et al MrBayes tgMC(3): a tight GPU implementation of MrBayes. PLoS One. 2013;8(4):e60667 Epub 2013/04/18. 10.1371/journal.pone.0060667 23593277 PMC3621901

[49] Whelan S, Goldman N. A general empirical model of protein evolution derived from multiple protein families using a maximum-likelihood approach. Mol Biol Evol. 2001;18(5):691–9. Epub 2001/04/25. 10.1093/oxfordjournals.molbev.a003851 . 11319253

