# Supplementary figures and images for: Episodic evolution of a eukaryotic NADK repertoire of ancient provenance
Source: PLoS One. 2019 Aug 1;14(8):e0220447. doi: 10.1371/journal.pone.0220447 (PMC6675116; doi:10.1371/journal.pone.0220447)

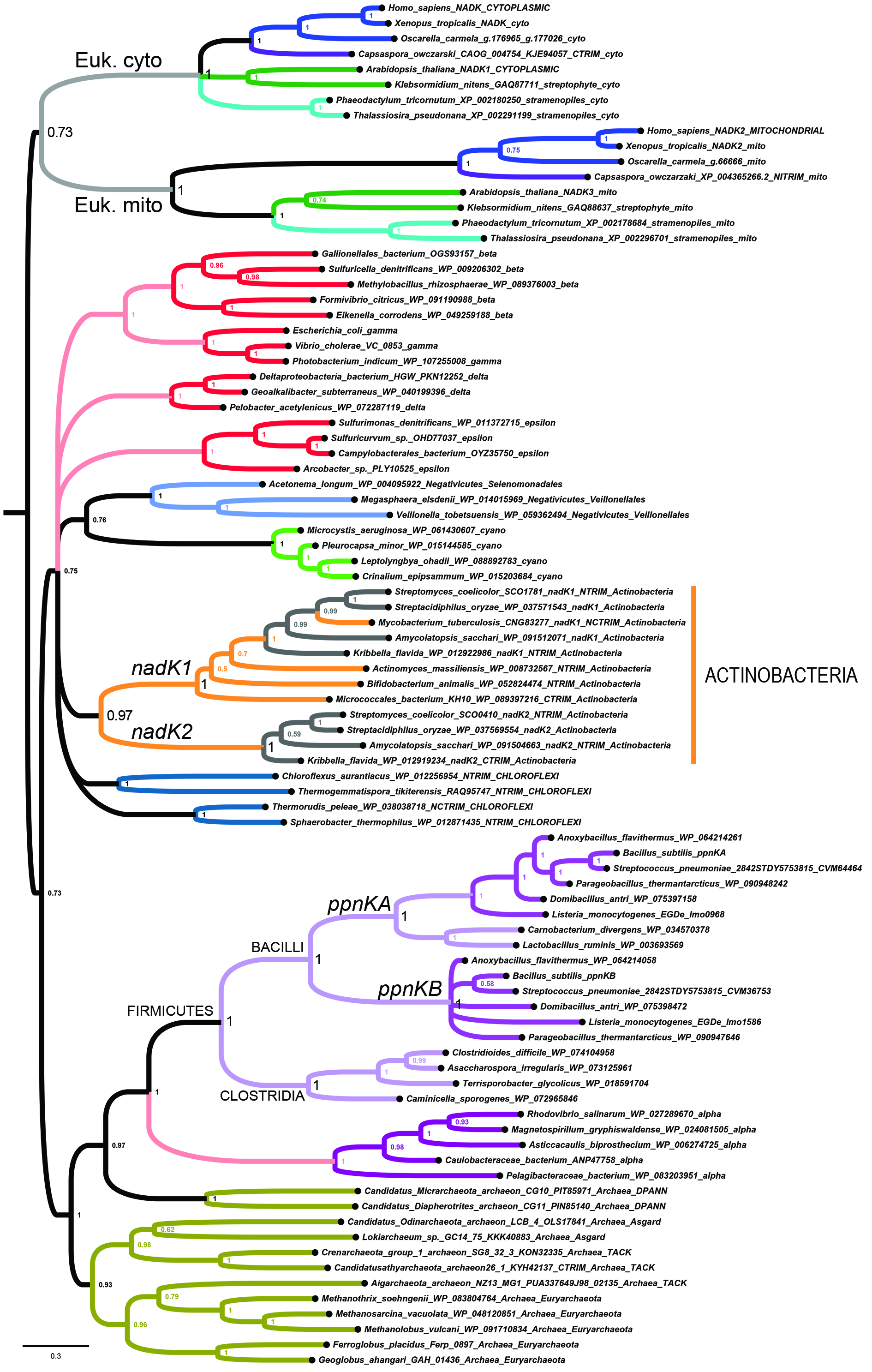

Supplement: S1 Fig — (TIF) [file pone.0220447.s001.tif]

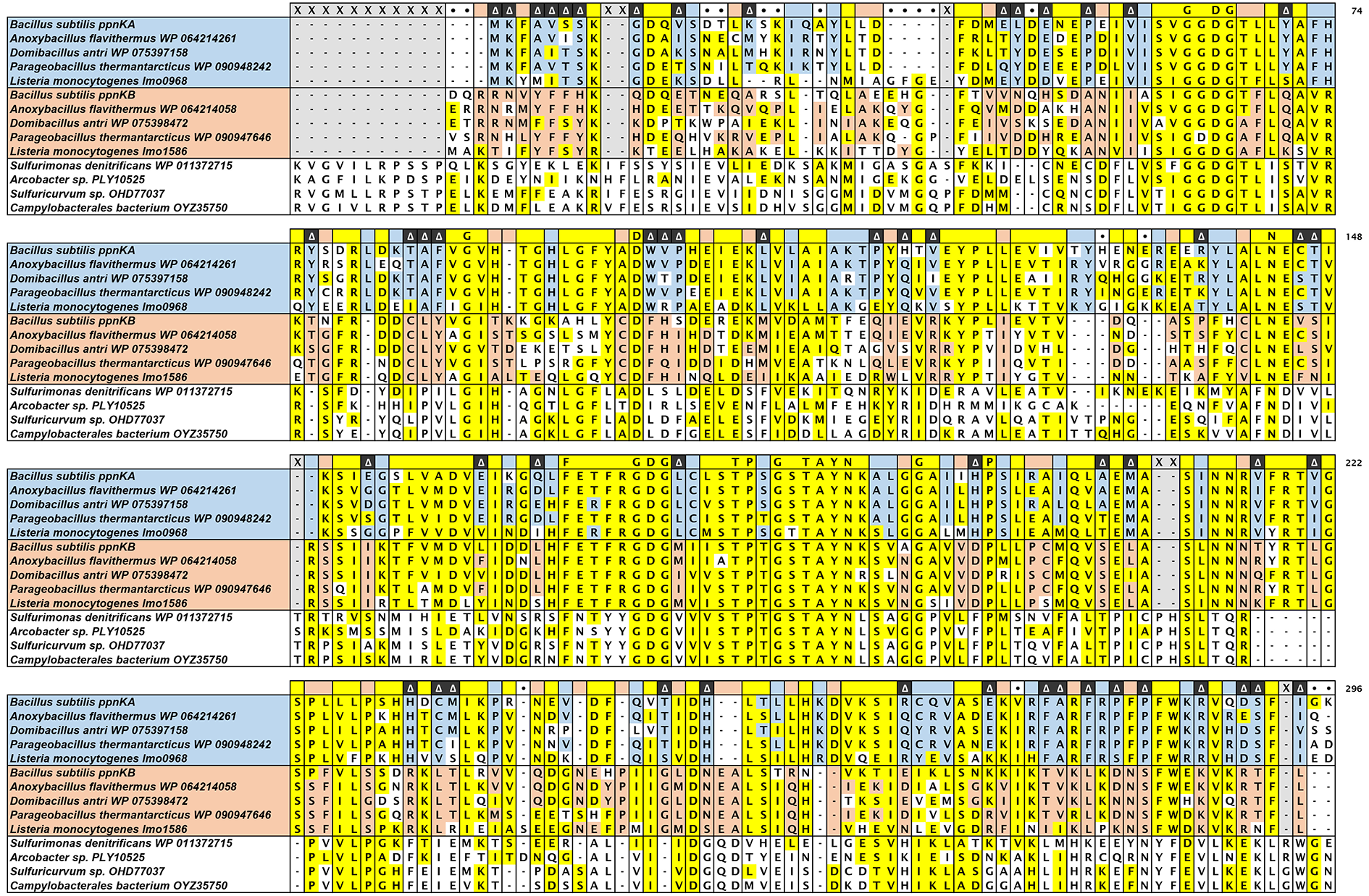

Supplement: S2 Fig — The amino acid residues that are differentiated in PpnKA or PpnKB are indicated in blue and red, respectively, while residues that are widely conserved are indicated in yellow. (TIF) [file pone.0220447.s002.tif]
