# Supplementary material for: Episodic evolution of a eukaryotic NADK repertoire of ancient provenance
Source: PLoS One. 2019 Aug 1;14(8):e0220447. doi: 10.1371/journal.pone.0220447 (PMC6675116; doi:10.1371/journal.pone.0220447)
Supplement: S1 Table — (DOCX) [file pone.0220447.s004.docx]

**S1 Table. Proposed names for *NADK* genes in *Drosophila* and *Caenorhabditis***

| **Model genetic organism** | **Current systematic gene name** | **Name based on orthology to human** | **Eukaryotic gene clade (sub-clade)** |
| --- | --- | --- | --- |
| *D. melanogaster* | *CG6145* | *NADK1a* | “cyto” |
| *D. melanogaster* | *CG33156* | *NADK1b* | “cyto” |
| *D. melanogaster* | *CG8080* | *NADK2* | “mito” |
| *C. elegans* | *Y17G7B.10* | *NADK2a* | “mito” (nematode “nmito”) |
| *C. elegans* | *Y77E11A.2* | *NADK2b* | “mito” (nematode “m2c”) |
