# Supplementary material for: Episodic evolution of a eukaryotic NADK repertoire of ancient provenance
Source: PLoS One. 2019 Aug 1;14(8):e0220447. doi: 10.1371/journal.pone.0220447 (PMC6675116; doi:10.1371/journal.pone.0220447)
Supplement: S2 Table — For each analysis shown, we include the FASTA file (“.fas”), the sequence alignment file (“.masx”), the nexus file (“.nexus”), and associated run files. Each set of files shares the same base file name indicated in the table. Line breaks in FASTA sequences typically indicate places where sequences were trimmed. Some hand curated sequences are documented in WORD files with the base name “CURATION”. All file are included as a compressed archived (“S1 File.tar”). (DOCX) [file pone.0220447.s005.docx]

**S2 Table. List of supplementary files associated with phylogenetic analyses.**For each analysis shown, we include the FASTA file (“.fas”), the sequence alignment file (“.masx”), the nexus file (“.nexus”), and associated run files. Each set of files shares the same base file name indicated in the table. Line breaks in FASTA sequences typically indicate places where sequences were trimmed. Some hand curated sequences are documented in WORD files with the base name “CURATION”. All file are included as a compressed archived (“File_S1.tar”).

| **Fig.** | **Base file name** | **Number of Metropolis-coupled generations** | **Average standard deviation of split frequencies** |
| --- | --- | --- | --- |
| **1** | NADK_v57 | 800,000 | 0.011 |
| **2** | NADK_v59-fungi-OV | 641,000 | 0.017 |
| **3** | NADK_v63-rando-protists-NCLIP | 1,200,000 | 0.033 |
| **4** | NADK_v58-nematodes | 2,100,000 | 0.031 |
| **5** | NADK_v58-nematodes_only | 1,200,000 | 0.007 |
| **6** | NADK_v62-holozoa (OV) | 1,200,000 | 0.028 |
| **7** | BACILLI_NADK_v108 | 300,000 | 0.009 |
| **S1** | NADK-comparison_v260 | 1,200,000 | 0.018 |
